# Experiences of employees with arm, neck or shoulder complaints: a focus group study

**DOI:** 10.1186/1471-2474-15-141

**Published:** 2014-04-29

**Authors:** Nathan Hutting, Yvonne F Heerkens, Josephine A Engels, J Bart Staal, Maria WG Nijhuis-van der Sanden

**Affiliations:** 1 Department of Occupation and Health, HAN University of Applied Sciences, P.O. Box 6960, 6503 GL Nijmegen, The Netherlands; 2 Radboud university medical center, Scientific Institute for Quality of Healthcare, Nijmegen, The Netherlands

**Keywords:** CANS, Employee, Patient perspective, Work-related upper extremity disorders, WRUED

## Abstract

**Background:**

Many people suffer from complaints of the arm, neck or shoulder (CANS). CANS causes significant work problems, including absenteeism (sickness absence), presenteeism (decreased work productivity) and, ultimately, job loss. There is a need for intervention programs for people suffering from CANS. Management of symptoms and workload, and improving the workstyle, could be important factors in the strategy to deal with CANS. The objective of this study is to evaluate the experienced problems of employees with CANS, as a first step in an intervention mapping process aimed at adaptation of an existing self-management program to the characteristics of employees suffering from CANS.

**Methods:**

A qualitative study comprising three focus group meetings with 15 employees suffering from CANS. Based on a question guide, participants were asked about experiences in relation to continuing work despite their complaints. Data were analysed using content analysis with an open-coding system. During selective coding, general themes and patterns were identified and relationships between the codes were examined.

**Results:**

Participants suffering from CANS often have to deal with pain, disability, fatigue, misunderstanding and stress at work. Some needs of the participants were identified, i.e. disease-specific information, exercises, muscle relaxation, working with pain, influence of the work and/or social environment, and personal factors (including workstyle).

**Conclusions:**

Employees suffering from CANS search for ways to deal with their complaints in daily life and at work. This study reveals several recurring problems and the results endorse the multi-factorial origin of CANS. Participants generally experience problems similar to those of employees with other types of complaints or chronic diseases, e.g. related to their illness, insufficient communication, working together with healthcare professionals, colleagues and management, and workplace adaptations. These topics will be addressed in the adaptation of an existing self-management program to the characteristics of employees suffering from CANS.

## Background

Many people suffer from complaints of the arm, neck or shoulder (CANS). The reported point prevalence ranges from 1.6%-53% and the 12-month prevalence from 2.3%-41% depending on the setting, definition, and classification used [1-3]. CANS is persistent; 77% of employees with CANS reported to still have complaints after six months [4].

Although CANS is common, no international consensus has been reached concerning related terminology [5]. However, in the classifications of CANS, a distinction is usually made between specific CANS (such as epicondylitis and carpal tunnel syndrome) and nonspecific CANS [6].

CANS causes significant work problems, including absenteeism (sickness absence), presenteeism (decreased work productivity) and, ultimately, job loss [7,8]. In the Netherlands, CANS is responsible for 15% of the total number of sick days [9] and the total annual costs for people with CANS are estimated at 2.1 billion euros due to medical expenditure (direct costs) plus decreased productivity, sick leave, and chronic disability (indirect costs) [10].

Although the exact etiology of nonspecific CANS remains unknown, it is presumed to have a multifactorial origin [11-14]. Physical characteristics (e.g. wrong working posture), psychosocial characteristics (e.g. lack of social support from colleagues and/or management), personal factors (e.g. an ineffective approach to stress management, an adverse workstyle) of the individual worker, as well as characteristics of their work environment (e.g. facilities, work culture), contribute to the development and persistence of these complaints [4,11-16]. The importance of each factor, and its individual contribution to the risk profile, varies between individuals and work environments [16].

There is conflicting evidence regarding the effectiveness of ergonomic interventions [17-21]. Nowadays, multi-component interventions that include both biomechanical as well as psychosocial components are recommended [13,18,22]. A workstyle intervention introduced by Bernaards et al. [23,24] among computer workers focused on behavioral change with regard to body posture, workplace adjustment, breaks, and coping with high work demands. Compared with usual care, the intervention was found to be effective in improving recovery from neck/shoulder symptoms and reducing pain on the long term (12 months), whereas no effects were found after six months or for pain in the arm/wrist/hand.

Among Dutch employees with sickness absence due to CANS, 24% believes that work is the main cause of their complaints and 30% stated that these complaints are partly caused by work [9]. Also, 19% of the Dutch employees stated that measures at work are needed for CANS because facilities are either not, or are insufficiently, available [9].

Self-management is an increasingly used approach in chronic illness care to improve self-efficacy (described as beliefs in one’s own capability to organize and execute the courses of actions required to reach one’s goals), and wellness behaviors (behavior leading to a healthier way of living) [25-27]. Barlow et al. defined self-management as “*the ability to manage the symptoms, treatment, physical and psychosocial consequences, and lifestyle changes inherent in living with a chronic condition”*[28]. Self-management programs aim to help participants make informed choices and then carry them out [26]. Self-management interventions focus primarily on encouraging patients to be involved with and in control of their own treatment, as well as improving their understanding of how their condition and treatment affect their lives [29]. As a result, self-management interventions reflect a change from a patient passively receiving care to a collaborative model in which the patient and provider share their knowledge and work together to achieve optimal self-management [29].

There is a need for intervention programs for people suffering from CANS [6,15,16]. Management of symptoms and workload, and improving workstyle, could be important factors in the management of CANS. In their intervention, Bernaards et al. mainly focused on the physical factors of workstyle [23,24], whereas self-management programs also focus on psychological characteristics, personal factors and characteristics of the work environment. Moreover, the participants are asked to set targets: Specific, Measurable, Acceptable, Realistic, Time-bound (SMART), which then are formulated in terms of behavior. In addition, action plans are made.

Detaille et al. developed a self-management program for employees in the Netherlands with a chronic disease; results showed that, for the intervention group, the attitude towards self-management at work (enjoyment) improved after 8 months [30,31]. Our aim is to adapt that program for use among employees with CANS, and add an ehealth component following the process of intervention mapping (IM), which is a staged process used to develop evidence-based and context-relevant health promotion or injury prevention programs [32]. The ehealth component has been added because of the multifactorial origin and diversity of the symptoms of CANS: by adding an ehealth component, part of the subgroup-specific related information can be provided in a tailored way (in which participants can make their own choices). In this way, the time during the meetings can be used more effectively and the information is available at every moment. The present study focuses on the first stage of IM in which the problem is identified and the intervention context is investigated [33]. This phase is crucial to understand the end-users’ perspective in order to determine the intervention content and to increase the likelihood that the strategies will be adopted and implemented [32].

Aim of the present study is to identify the problems as experienced by employees with CANS. With this information, the existing self-management program of Detaille et al. [30] can be adapted to specifically fit the characteristics and needs of employees with CANS.

## Methods

### Study design

In 2012, three focus group meetings were held among employees with CANS; all sessions took place at the HAN University of Applied Sciences (Nijmegen, the Netherlands). The Radboud university medical center medical ethic committee declared (registration number 2013/317) that the study does not fall within the Dutch law on ‘Medical Research involving Human Subjects’ (the WMO) and that therefore, for performance of this research, no approval is required from a medical ethic committee. The research protocol fulfilled the criteria of the Declaration of Helsinki - Ethical Principles for Medical Research Involving Human Subjects.

We used focus groups to investigate the range of ideas that people have about a certain topic; such groups can uncover factors that influence opinions, behavior or motivation [34]. Focus groups can be used in program development and have proven helpful in the needs assessment, mostly because they provide an interactive environment in which ideas can emerge from the group [34]. A group possesses the capacity to become more than the sum of its parts, and to exhibit a synergy that individuals alone do not possess [34]. Therefore, focus groups were considered to be the most suitable method in view of the aim of this study, i.e. to identify the problems (at work) as experienced by employees with CANS.

### Participants

A purposive, homogeneous sampling technique was used to identify potential participants. Participants were recruited from the staff of the HAN University of Applied Sciences and the Radboud university medical center (both located in Nijmegen, the Netherlands). Participants were recruited via electronic occupational news mails and informed about the research project by occupational health staff. Generally, self-management interventions focus on chronic conditions and, therefore, participants were only included if they had any complaints of the arm, shoulder and/or neck persisting for longer than 12 weeks, and if the complaints were caused or worsened by their job and/or limited their participation in work. The inclusion criteria used for the present study will also be used to include participants in the adapted self-management intervention for employees with chronic non-specific CANS. Each participant was informed that participation was voluntary and that data would be used anonymously. Employees fulfilling the inclusion criteria were asked to fill out a short questionnaire (demographics) prior to the focus group meeting.

All participants gave informed consent to participate in the study and to allow audio-recording of the sessions. All participants received a gift of 20 euro for their participation.

### Focus group meetings

Following the recommendations of Krueger and Casey [34] a question guide with open-ended questions was developed (Additional file 1). The content and the order of the different question categories were developed based on the recommendations of Krueger and Casey [34]. The selected topics were based on a recent multidisciplinary guideline for nonspecific CANS [21] and on the original self-management program as developed by Detaille et al. [30]. The selected topics ensured the multifactorial perspective of the focus group sessions. Each focus group session was moderated by the first author (NH) using a standardized script. The group members were asked about their experiences at work and their needs for continuing work despite their complaints. The topics included participants’ experiences with their complaints, experienced problems with work activities, dealing with work problems, support and help of others (at work and at home), and communication about their complaints. In addition, a healthy lifestyle was discussed. When the group discussion was not sufficiently facilitated by the question alone, or if the question was not clear enough, the moderator could give some examples. The moderator actively stimulated interaction and discussion between the participants. Finally, participants were asked what kind of information related to CANS they would like to receive and what they would like to learn if they would follow a self-management intervention.

All focus group sessions were audio-recorded and notes were taken by an assistant (LD). In each meeting the question guide was followed. The moderator made sure that every participant was involved in the discussions. Each session lasted about 120 min. Debriefing was performed after each session.

### Data analysis

The audio-recordings were transcribed by the assistant (LD). Member checks were performed after drafting the manuscript, one year after the focus group sessions. If no response to the first email was received from participants within 10 days, a reminder was sent by email. The first author (NH), trained in qualitative research methods, performed the data analysis. Data were analysed using conventional content analysis [35,36], which is generally used with a study design whose aim is to describe a phenomenon [35]. The aim of content analysis is ‘to provide knowledge and understanding of the phenomenon under study’ [37]. Content analysis has a long history in research and is used to analyze text data and can be used in analyzing focus groups [35].

After reading each transcript multiple times, the transcript was analyzed using content analysis with an open-coding system [36]. New codes were added when considered necessary. After this, the codes were sorted into categories based on how different codes are related and linked [35]. Then, the emergent categories were used to organize group codes into meaningful clusters [35], expressing the experiences of employees with CANS.

The Atlas.ti (version 7.082) program was used for analysis. During data analysis, the emerging themes were discussed in a small expert group (NH, YH, SD). Moreover, by reading all the transcripts, the expert group checked that no themes were missed. The supporting quotes related to each theme were discussed in the expert group.

## Results

Initially, 20 employees wished to participate; of these, two were excluded because they did not fulfil the inclusion criteria and three persons withdraw consent after obtaining more information about the study. Of the remaining 15 participants, three were interviewed individually as they were unable to attend one of the focus group meetings.

The mean age of the participants was 46.9 years and they worked in various professions within the organizations. Table 1 presents the characteristics of the 15 participants fulfilling the inclusion criteria and Table 2 presents the demographic profile of each participant. In general, the same issues emerged and were discussed in all three focus group meetings. In session three, no new issues were discussed and no new codes were added. All participants were successfully reached for the member checks. None of the participants indicated that our interpretation was not correct; no changes were made after the member checks. The topics that emerged during data analysis are described below.

**Table 1:** Characteristics of the study population (n = 15)

**Variables**	**Values**
Mean age in years, (range)	46.9 (25–56)
Male, n (%)	1 (6.7)
Female, n (%)	14 (93.3)
Mean number of work days per week (range)	4.1 (2.4-5.0)
Mean hours of work per week (range)	30.7 (18–50)
Mean hours of PC work per day (range)	4.4 (0.5-8.0)
Education level, n (%)	
*Preparatory secondary vocational education*	1 (6.7)
*Senior secondary vocational education*	3 (20.0)
*Higher professional education*	7 (46.7)
*Academic higher education*	4 (26.7)
Mean disability score on work (1–10) (range)	3.8 (0–7.0)
Complaints, n (%)	
*Hand*	3 (20.0)
*Wrist*	3 (20.0)
*Under arm*	2 (13.3)
*Elbow*	1 (6.7)
*Upper arm*	4 (26.7)
*Shoulder*	13 (86.7)
*Neck*	12 (80.0)
Duration of complaints in weeks, (range)	222 (20–936)
Side of complaints, n (%)	
*Left*	2 (13.3)
*Right*	7 (46.7)
*Both sides*	6 (40.0)

**Table 2:** Demographic profile of the study population

**Participant ID number**	**Gender**	**Age (years)**	**Education level**	**Profession**	**Organization**	**Hours of work per week**	**Hours on PC per day**	**Body region of complaints**	**Duration of complaints (weeks)**	**Disability score on work (0–10)**
1	Female	56	AHE	Lecturer, supervisor, coach	HAN	26	2.0	Shoulder, neck	20	6
2	Female	44	PSVE	Secretary	HAN	32	6.0	Wrist, lower arm, shoulder, neck	52	2
3	Female	44	SSCE	Administrative assistant	HAN	18	4.0	Hand, elbow, upper arm, shoulder, neck	104	4
4	Female	51	HPE	Senior analyst IVF laboratory	RUMC	32	2.0	Lower arm, upper arm, shoulder, neck	104	2
5	Female	48	AHE	Research coordinator	RUMC	36	6.0	Shoulder, neck	780	3
6	Female	54	HPE	Intensive care nurse	RUMC	20	2.0	Shoulder	936	4
7	Female	46	HPE	Nurse	RUMC	32	2.0	Shoulder, neck	31	7
8	Female	55	HPE	Outpatient assistant	RUMC	28	8.0	Upper arm, shoulder, neck	104	0
9	Male	44	SSCE	Security officer	RUMC	40	6.5	Hand, wrist, shoulder, neck	400	6
10	Female	56	HPE	Intensive care nurse	RUMC	32	3.0	Hand, upper arm, shoulder, neck	416	5
11	Female	47	SSCE	Senior sterilization employee	RUMC	32	0.5	Shoulder, neck	30	3
12	Female	25	AHE	PhD student	RUMC	27	6.0	Wrist, neck	104	3
13	Female	42	HPE	Analyst	RUMC	32	6.0	Shoulder	70	7
14	Female	55	AHE	Pharmacist / PhD student	RUMC	50	5.5	Shoulder	76	3
15	Female	37	HPE	Lecturer, trainer	RUMC	24	6.0	Shoulder, neck	104	0

PSVE = Preparatory secondary vocational education, SSCE = Senior secondary vocational education, HPE = Higher professional education, AHE = Academic higher education, HAN = HAN University of Applied Sciences, RUMC = Radboud university medical center.

### Ideas about the causes of complaints

Causes of complaints vary between participants. Some employees stated that the cause of their complaints is mainly physical, e.g. hereditary, or (partly) caused by an underlying condition such as diabetes mellitus. Some participants have ‘weak muscles or tendons’ or their complaints are caused by continuous contraction of the muscles. Workload in the past, or in the current job, was also mentioned as a possible cause of complaints. Some participants were uncertain about the cause of their complaints.

At work, trying to meet expectations and maintaining a high level of standards can result in stress and taking insufficient time for breaks. Both these are mentioned as aggravating factors and a possible cause of complaints. One participant said:

What I’ve encountered at work on several occasions when the pressure and the workload were too high, is that my physical complaints increase very quickly. (Participant 6)

Stress and related muscle tension are reported to be a major trigger of symptoms. For example, one participant stated:

For me, stress is a major trigger. If I’m stressed at work during the day - I have neck pain that evening. (Participant 6)

In addition, prolonged working in a wrong posture, e.g. on a computer (especially a laptop), as well as lack of alternation in work activities during the day, are mentioned as aggravating factors. Also, complaints are worsened by other sub-optimal working conditions and prolonged concentration on work tasks; one participant stated:

In fact it’s the cause of my complaints. Remaining in one specific position for a longer time, particularly when I’m sitting behind the microscope and working in a focused way - I have the tendency to tense my neck muscles. (Participant 4)

### Dealing with non-visible complaints

Participants often find it difficult to deal with the lack of understanding they may experience from others. Generally speaking, their colleagues and/or managers seem unable to easily observe that someone is in fact suffering from CANS. One participant said:

People don’t notice that someone is sick or if something is wrong. It’s better to break a leg! If you’re walking with crutches, the door is certainly held open for you. But now, they don’t notice anything about you. (Participant 9)

It is normal for employees to appeal to their colleagues (whether or not they have CANS) for various types of assistance. Participants find it difficult to say ‘no’ to these requests and to explain that they suffer from CANS. On the other hand, some participants mention that the advantage of having a ‘non-visible’ complaint is that this avoids being asked lots of questions about the complaints during the day.

### Experiences with different forms of treatment

Most employees have tried various forms of treatment such as physical therapy, manual therapy and exercise therapy. In the case of physical therapy, treatment sometimes consisted of local treatment of the painful area and/or exercise. Many participants have also consulted their general practitioner and, in a few cases, a medical specialist (e.g. an orthopaedic surgeon, rehabilitation physician or rheumatologist). Occasionally the complaints were treated with injections. Within the organization, many employees have consulted the occupational physician, occupational health staff, or physical therapist. Some participants have used the possibilities for workplace adjustment(s) and chair massage. Although all participants still suffered from CANS, their experience with care was mainly positive. However, in some cases it was difficult to find the appropriate healthcare professional, as one participant stated:

… the family doctor was repeatedly referring me to an orthopaedic surgeon who only wants to operate, and that’s pointless in my opinion. Therefore, on each occasion, that was a dead-end street. (Participant 12)

### Workplace adjustments

For some employees, a workplace investigation was performed by the occupational health staff and adjustments to the workplace were made - as one participant stated:

They made modifications to the work station: my desk was too high and that’s been adjusted to my height… and they made sure that my computer screen was at eye level. (Participant 5)

Some participants use adjustments, such as a writing tablet, voice recognition software, or repetitive strain injury software. One employee said she found it difficult to request adjustments because these were charged to the department budget. Very few participants used a brace for support.

Moreover, many participants experience problems with making (physical) adjustments to their workplace. In many cases the workplace cannot be properly adjusted, e.g. the computer monitors are too high, the chairs are not (properly) adjustable, or some doors are very difficult to open. In general, people have many problems with the construction and/or the furnishings of some buildings. Participants can become frustrated about this - as one person said:

… as with many important things - such as the distance from the computer screen - that is definite and is often not adjustable. (Participant 8)

Even when adjustments are possible, in many cases the workstations are multifunctional, e.g. if an employee does not have his/her own personal workplace, then customized adaptation is not possible. Also, in many cases participants can only adjust the seat height and little else. One participant remarked:

… provide me with at least seven adapted chairs - because I sit everywhere. Also - provide me with seven computer screens that can be placed in a lower position. (Participant 10)

Information is required about the work environment related to CANS. Adjustments at the workplace and use of shortcuts are recommended. If workplace investigations have not yet been performed, this is because the participants do not know what items they should examine, or do not know how to initiate a workplace investigation within the organization. This seems to be crucial information. Some identified needs of the employees focus more on working, e.g., working in a quiet environment because they cannot concentrate, working partly at home, or having more flexibility in their schedule at work; as one participant stated:

Flexible work - so you can get up once in a while and walk around. Flexible work hours. (Participant 14)

### Available information about complaints

Some participants indicate that because they have long-lasting complaints, they have sufficient knowledge about their complaints. However, participants stated that for employees with a shorter duration of complaints basic information is needed about the complaints, including causes and possible solutions. Participants would like specific information about possible treatments and an overview of treatment options within the organization. Also, more general information about muscle relaxation (including exercises) is required. Participants are also interested in the psychological components of CANS and of pain in general. One participant stated that she changed her opinion about her pain when she understood that her pain was not a signal related to tissue damage, she remarked:

I handle my pain completely differently now. Pain that isn’t followed by anxiety that possibly leads to even more suffering is much easier to treat. Your perception of pain makes a big difference. (Participant 5)

In conclusion, information about working posture and tips related to office work need to be addressed in intervention programs. Also, there seems to be a need for information about a variety of topics, such as exercises and psychological components of CANS.

### Work-home balance and fatigue

Some participants report a lack of balance between their work and private life. At the end of the day they feel exhausted. In two of the three focus group meetings, fatigue emerged as a major issue. Fatigue increases during the day, participants sleep badly due to pain, and are often tired the following day. This becomes a vicious circle with fatigue having a negative impact on work performance and on concentration levels during the day. Thus, fatigue seems to play a major role in the life of most of the participants. The following was stated by one participant:

I find the feeling of tiredness extremely bothersome. Your concentration is then not 100% - you have to check what you’re doing three times over, in my case that I have not mixed up the patients. You’re very aware that your feeling of fatigue increases as the day progresses. (Participant 14)

### Coping with complaints

Participants find it difficult to deal with their complaints. This is mainly because they suffer from ‘nonspecific’ complaints for which no clear solution is available. In the present study, participants generally found it difficult to manage prolonged work activities and to take sufficient breaks, and needed to pay sufficient attention to their physical posture at work. It was said to be challenging to find a balance between all the requirements related to activities at work, e.g. to avoid physical overload. Alternating between different types of work activities is not always possible. In addition, dealing with ongoing pain is difficult and pain often limits the level of performance of work activities.

One participant said:

For example, it’s also a nuisance on a day when I’m interacting with many people. My processing capacity is limited due to my chronic neck pain. (Participant 1)

Participants tend to accommodate themselves to the complaints, which in some cases, makes the complaints more manageable. Some participants stated that it is important to accept that one has physical complaints. Nevertheless, they are still often confronted with their complaints in daily life, e.g. when picking something up, or simply when putting on a coat. Learning how to deal with the complaints and accepting them are considered to be important.

Participants tried to reduce the impact of their complaints in several ways, e.g. by making adaptations in various areas. They tried to reduce their physical load in general or during their work. Some also tried alternative tasks and paid more attention to their posture whilst working. Some participants started looking for other work or different types of work tasks.

I wanted to do something else, something more in the direction of education. However, my physical complaints played a role. I thought: I’m so young and have such a heavy burden of complaints, it would be better to change now. (Participant 13)

Other participants made adjustments in their planning of tasks and work schedule, and some decided to reduce their number of working days - as stated by one participant:

Eventually I did choose to work fewer hours - because I was simply no longer capable of working fulltime. (Participant 14)

Participants also stated that they tried to increase their understanding about their complaints and about the causes of their complaints. This awareness and reflection on their own situation were experienced as meaningful and are considered to be important skills. Other participants focused on other aspects, as stated by one participant:

I didn’t make a serious effort to organize another workstation because I wasn’t convinced that this should be my first priority. First, I have to try and improve my capacity as much as possible through better training of my muscles, or relaxing my joints. (Participant 13)

Some participants tried to influence their complaints through sports and exercises, and tried to upgrade their physical capacities. On the other hand, some participants stopped stressful sports activities because they thought these might aggravate their complaints. Although the importance of exercises is generally recognized, participants find it difficult to perform exercises over a long period, and mainly perform exercises at the moment they have more severe complaints.

Several participants were involved in running/walking, swimming, cycling, aerobics, or shooting sports. A few participants stated that their complaints had worsened when performing fitness training. Also, having too little time was a reason not to perform sport activities. In general, most participants recognize the importance of fitness training - as stated by one participant:

I have the idea that if I hadn’t stopped my weight training (strength training) program then perhaps I would not have any physical complaints. (Participant 14)

Participants stated that in their spare time sufficient relaxation and time-off are important: some benefitted from the application of heat, a visit to the sauna, or yoga exercises.

### Coping with workload and stress

Participants indicate that in recent years the workload has increased. For example, in one institution, due to financial cut-backs there is a hiring freeze; however, because there is more work and some colleagues might be on sick leave, the work accumulates. Moreover, the physical distances within an organization have often increased due to rebuilding, and an increasing number of activities have to be registered. All of these activities involve considerable time and increase workload stress. One participant stated:

You have to be able to prove that you’re the best hospital. Or that you have the best ratings … and these are only obtainable through registration, registration, registration. Therefore, you have to be able to justify almost everything that you do, and you have to register this, and you are also very aware of this. However, all this extra work often costs me more energy. (Participant 10)

Due to the increased workload participants perceive that there is no time to read emails during work time. Also, participants indicate that there seems to be insufficient/no time to take a break. All this causes stress at the workplace and gives the impression that one’s leisure time is being swallowed up by work; one participant stated:

Before - I could still sometimes read emails during my office hours - but that’s no longer possible. … if I look at my emails at home during the evenings, then I see 12 mails, with attachments – read this, read that. Then I ask myself - what do they want? We always have to do the training and take exams in our own time. This is in addition to the enormous pressure at work that you already have. (Participant 8)

According to most participants, there is little opportunity for flexibility. For example, since work activities tend to be increasingly specific, it is difficult to change shifts. There is practically no possibility to influence one’s work schedule, which increases the workload and stress. Due to this workload and stress, less attention is paid to maintaining a good physical posture and this can cause the CANS to recur faster. Participants find that it is not always easy to deal with the stress and pressure of work. Some participants have almost given up and just accept things - one participant stated:

At the moment I no longer have so many problems with stress at work - it’s extremely busy, but 16:00 will come around anyway - and I can’t do anything except to work. (Participant 7)

Ensuring adequate relaxation, having sufficient discipline, and creating enough time for tasks/exercises is also considered important by the participants. All of these seem to be important skills.

The culture within an organization also plays a role. Often participants perceive that there is less time to complete the work, although the employer expects participants to complete their work. Generally speaking, employees are not expected to be absent due to CANS. For example, it is acceptable for someone with a temperature of 40°C to stay at home, whereas for an employee with CANS the situation is different and they find it difficult to stay away from work. Moreover, participants stated that when an employee with CANS is at work he/she is expected to be 100% employable, which is often not the case. One participant stated:

… and when you’re at work, they only think in terms of whether you’re there or not there… and if you’re there, then they think that everything is alright. This applies even though you’re often walking around at work in a lot of pain. (Participant 5)

Several employees are aware of the financial restrictions within the organization, which is a source of frustration. In many cases participants experienced that insufficient or no financial resources are available to make the required (physical) adjustments to the workplace, e.g. an adjustable desk. This, and a certain level of bureaucracy, is illustrated by a work situation where some hooks were placed too high for an employee:

… so I asked whether I can hang these myself, so that I can feel more comfortable - but that’s not allowed. That has to be decided again by a committee, because everything has to be the same everywhere. Then it became ten times more expensive … and then they said it’s impossible because the costs are too high! (Participant 10)

### Setting limits

Some participants do set a limit for themselves, or ask for help if they can no longer handle the work themselves. However, some have a problem with setting their individual limits: as one participant remarked:

I find it very difficult to set these limits - you want to do your work as well as you can, you really want to do everything that’s asked of you. (Participant 5)

In general, participants seem to continue working for too long with their complaints without taking any action. Participants indicate that it is difficult for them to set limits in an early stage and this could be an aggravating factor.

Taking into account one’s own limits, but also realizing one’s own advantages, is considered important. One participant had a practical solution for the prevention of stress:

Many people are extremely busy at work, walking in and out; I now have a ‘Do not disturb’ sign hanging on my door. This works really well on days when it’s really busy and I set the ‘Do not disturb’ sign in red; then I can concentrate on my work. (Participant 4)

### Support from others

Participants do not always find it easy to talk about their complaints and/or to bother others about their problems. They do not want to complain, not even to healthcare professionals. Generally there are no major problems with communication, but explaining the type of pain is sometimes difficult.

Most participants experienced sufficient support from colleagues (although a few experienced no support from colleagues). Support is sometimes interpreted as help/support with work, and sometimes as a sympathetic ear and/or ‘mental’ support. One participant stated:

If I let anyone know that I’m having problems, then my colleagues are very considerate or want to take over some of my workload. But because my problem is not always so evident, I’m not continuously being helped by my colleagues. I have to be the one to let them know - but then I do receive understanding and support. (Participant 4)

Participants do talk with their colleagues about their complaints, especially if others also have physical problems. However, not everyone feels the need to talk about their problems at work, as one participant remarked:

I don’t talk much about it at my work unless it becomes a real problem. Up to that point, I just continue doing what I have to do … I do discuss it once I’m home. (Participant 12)

Thus, some of the participants prefer to keep their problems to themselves and only talk about their complaints with colleagues or a supervisor if they really have to.

Most of the participants received sufficient support and interest from their supervisor; one person remarked:

I certainly have that, in fact one supervisor recently asked how I was really doing: “I don’t see you very often with the brace, are you OK?” And then it’s certainly noticeable when I’m walking around with that thing, or not. That lets everyone know whether I am doing OK or not - that’s a pleasant feeling. (Participant 4)

However, some participants experienced insufficient or no support. In those cases the supervisor seems to be more concerned with the overall state of the department than with how an employee with CANS can be supported. One participant said:

I don’t receive any support - because my manager is not present. And if she comes by, what will I say - she never stops walking. And if you say something, it’s “OK” (and she continues on her way), and it’s not worth the effort to have three discussion points, because she’s already gone after the first. (Participant 11)

All participants experience adequate support at home. If a family has some experience with similar problems, then family members can empathize with the situation. Regarding support at home, one person stated:

Yes absolutely, my husband regularly gives me hell in terms of “what are you doing now?”… He proposed to lower the desk and table and to try to work more with hotkeys - this has helped a bit. (Participant 3)

### Asking for help and support

Most participants have a relatively high threshold before asking for help and some participants think they should reduce this threshold for asking for help. Some participants do not ask for help because they want to stay ‘in control’ as long as possible. Generally, most participants set high standards and expectations for themselves. Participants who do ask for help usually get it, but sometimes feel burdened by it. People try to find alternatives or perform the tasks at another time, rather than asking for help. One participant stated:

I’m not quick to ask for help because I think I can come up with all sorts of tricks to solve it in another way. If I do ask for help, then it really is needed - and then people provide it without any problem. (Participant 4)

Sometimes participants do ask for help, but if support is perceived as not immediately available they tend to do the work themselves because, generally speaking, they think it cannot wait. Moreover, sometimes they want to do tasks again because their colleagues’ work does not meet their own standards, sometimes resulting in additional work and/or an angry client.

Some of the participants realize that it can be detrimental to do all the work themselves, to avoid asking for help, and to control things themselves; this often causes stress and aggravates complaints.

Participants consider social support at work and at home important. One participant said:

… and also if my supervisor can’t help, I still want her to support me. Especially to listen to me, this is the most important. (Participant 3)

Some find it difficult to ask for support, whereas others find it easy to ask for support or do not need it. Although no major problems with communication were experienced, communication skills can help with asking for help or support.

Participants consider the exchange of experiences with others and informing colleagues at work about the complaints as important. Although generally there are no major problems encountered with communication, participants stated that providing communication tools for discussion with colleagues/supervisors about their complaints is important.

## Discussion

To our knowledge this is the first study to investigate the experiences of employees suffering from CANS. In both their daily life and at work, employees suffering from CANS are faced with the challenge to deal with their complaints. The present study indicates that participants do not always have sufficient insight into the causes of complaints, and are not always fully aware of the possibilities to influence their complaints and of their own role in coping with their complaints. Generally, all participants suffered from pain and feel that they cannot manage this adequately. Some participants are aware that they have a problem with taking their own limits into account, while others often approach/go beyond their individual limits because they have a relatively high threshold before asking for help. Many participants feel that fatigue has a serious impact on their daily life and the management of their complaints. They feel uncomfortable about dealing with various disrupting physical factors (pain, disability, fatigue), psychosocial factors (stress, lack of balance work/private life, difficulties in communication, misunderstanding from others), personal factors (difficulties in setting limits, high threshold before asking for support, high level of personal standards and expectations) and environmental factors (non-optimal workplace, culture within the organization). All these factors should be addressed in future interventions.

The identified needs of participants include information about possible causes of CANS and possible solutions (e.g. treatment, facilities); (relaxation) exercises; working and dealing with pain, limitations, fatigue, workload and stress; work(place) adaptations; workstyle; taking into account one’s own limits and asking for help; communication with others; and awareness of one’s own advantage. Tools for dealing with these factors should be provided.

Although the etiology of CANS is multifactorial, most participants in the focus groups mention physical factors as the primary cause of their problems. Although this can indeed be the case, people may not be fully aware of the contribution of other factors in the etiology of their problem. Although psychosocial, personal and environmental factors are also mentioned, this is generally more in the sense of more aggravating factors.

The fact that CANS is a ‘non-visible’ complaint has various consequences. On the one hand participants indicate that this may contribute to their feeling of not being well understood whereas, on the other hand, it prevents colleagues from asking questions during the day. Thus, it seems that at least some participants find it difficult to communicate about their complaints. Moreover, if colleagues are not aware of the complaints, asking for help and obtaining social support may seem to be even more difficult.

Employees suffering from CANS are often confronted with a wide range of problems. Although most participants have taken many steps in an attempt to reduce their complaints, which vary from workplace adaptations to different types of (physical) therapies, they still have complaints and some are still looking for alternative treatment options. A few participants stated that their complaints had worsened when performing fitness training. Therefore, it seems important that people with CANS have sufficient knowledge and insight into the possible benefit and harm of sports activities, and that activities are well chosen and properly ‘dosed’. However, the awareness that there are opportunities for self-management differs between participants and most do not know how to cope with the working environment. Given the multifactorial origin of CANS, it was found that the variability between participants in taking into account all the possible contributing factors was relatively high.

In our study population the mean duration of symptoms was 222 weeks, indicating that most had suffered from these complaints for several years. This also implies that this group might be a useful source of relevant information for other employees with CANS in the a similar work environment, because they have experience in working with and finding solutions for their complaints. On the other hand, although most have tried various ways to reduce their complaints, the majority still suffer from CANS and still reported coping problems due to work environmental factors, to personal factors, and due to physical factors.

It should be noted that, because this study setting is rather specific and the participants relatively highly educated, the participants in this study are a specific group thereby making it difficult to generalize these results to other populations and to other settings. Therefore, the information gathered in this study will be used to select the most important topics for the self-management intervention; employees with CANS must be empowered to take control over their complaints in their work environment. The exact content of the identified topics may vary between different types of work settings.

The present study provides insight into perceptions and experiences of employees suffering from CANS and identifies a number of recurring problem areas. The results endorse the multifactorial (e.g. physical, psychosocial, environmental and personal) etiology of CANS [13]. Our results may help identify important areas that need attention in the treatment of employees suffering from CANS. This study identified several needs of employees with CANS. Insight in the symptoms of CANS and in its causal factors seems to be the first important point. Secondly, awareness and reflection on one’s own behaviors related to the working circumstances are considered important. Thirdly, participants need to develop their exercise, relaxation, coping, management and communication skills to deal with their problems on the long term. It is likely that knowledge and sufficient insight in the different causes of the complaints are important in order to raise awareness and reflection, and develop communication skills. All these items could be topics in the self-management intervention.

This study has several limitations. First, selection bias may have occurred regarding the study group as most participants were working in a hospital and, generally, have a long period of living with CANS. However, because participants in our study experienced some problems similar to those of employees with other types of chronic diseases, it seems plausible that these problems are also experienced by employees with CANS working in other settings. Moreover, we think that not (only) the work environment, but rather the personal characteristics of employees with CANS are (also) important when considering the causes of the complaints and when dealing with the complaints. However, this study was conducted in a healthcare and an educational setting, and the participants were relatively highly educated. Therefore, participants in our study group may be ‘better equipped’ to express themselves regarding CANS, due to the setting they work in and their higher level of education. Therefore, our results do not reflect the experiences of workers in different types of setting, such as factory workers.

Only one man participated; this is due to the larger proportion of woman working in the hospitals and the fact that women have a higher risk of developing CANS compared with men [38,39]. Moreover, we purposively selected participants based on some specific characteristics. We were interested in employees with complaints of the arm, neck and/or shoulder persisting for longer than 12 weeks. Moreover, the complaints must be caused or worsened by their job and/or limit their participation in work. Therefore, we purposively selected employees who met these criteria, using the described selection criteria. Because the aim of focus groups is not to infer but to understand, not to generalize but to determine the range, and not to make statements about the population but to provide insight into how people in the groups perceive a situation [34], the present results represent the experiences and perceptions of the participants of this particular study.

Moreover, three participants were interviewed individually as they were unable to attend any of the focus group meetings. This implies that these participants were not part of a group process and that, for these participants, the ideas did not emerge from the group. However, because these three participants wanted to participate and fulfilled the inclusion criteria, and all information about the experiences of employees with CANS was needed, we decided to perform interviews and analyze them together with the focus group results. Although this could have influenced the results, this does not seem to be the case, because no major differences in perceptions and experiences between participants of the focus groups and the interviews were identified.

The question guide was based on a recent multidisciplinary guideline for nonspecific CANS [21]. The question guide was also based on the original self-management program [30] in order to determine how the topics of the original program should be adapted. We assumed that some multifactorial aspects of CANS (physical characteristics; personal factors, e.g. stress management) would be mentioned and discussed by the participants themselves. Other topics (psychosocial characteristics, e.g. social support; the work environment, e.g. facilities; and some personal factors, e.g. asking for help) would perhaps need some more facilitation during the focus group. Therefore, these topics were individually addressed in the question guide to assist the moderator. Moreover, if new topics were introduced by the participants these were also facilitated. Due to the fact that the same issues were identified and discussed in all three focus group meetings and no new topics were introduced in the final session, it is highly likely that saturation was reached.

Another limitation is that, given the aim of this study (i.e. investigating the experiences of employees with CANS) and the multifactorial origin of CANS and many influencing factors, it was not possible to investigate all the topics and to extensively discuss all the emerging topics. We were mainly interested in the participants’ perception of the topics addressed in the question guide and therefore focused on topics fulfilling this aim.

Although member checking was performed, this took place one year after the focus group meetings. Therefore, it is possible that participants did not (exactly) remember the details of the focus group meetings. However, by providing the preliminary results of each session to the participants it seemed possible to check whether our interpretation of the data was correct; this was endorsed by the fact that none of the participants indicated that our interpretation was not correct.

Data were coded by one researcher. Multiple coding involves the cross-checking of coding strategies and interpretation of data by independent researchers [40]. However, the degree of concordance between researchers is not very important [40]; the main value of multiple coding is to supply alternative interpretations [40]. It is important that a transparent and systematic process is followed which can be carried out by one researcher, by a team, or by involving independent experts [40]. By discussing the emerging themes and looking for alternative interpretations in a small expert group, we addressed the potentially competing explanations.

In the present study, it is noteworthy that participants experienced some problems similar to those in employees with other types of chronic diseases [41,42]. Problems related to their illness, insufficient communication with supervisors, working together with healthcare professionals, colleagues and management, and adaptations at the workplace are considered important among employees with chronic somatic diseases [41,42]. Therefore, it seems plausible that a self-management intervention, including an ehealth module, covering these topics, and adapted to employees suffering from CANS with disease-specific information, may be effective in employees with CANS. Although there is inconsistent evidence for the effect of self-management programs for patients with chronic musculoskeletal pain [43-45], there is evidence that group-delivered short programs (<8 weeks) with a healthcare professional have the best potential [43]. In a recent study, a multi-component pain and stress self-management group intervention had better effects than individually administered physical therapy in the treatment of persistent musculoskeletal tension-type neck pain in terms of patients’ self-reported pain control, self-efficacy, disability, and catastrophizing over the 20-week follow-up [46].

The topics identified in the present study can contribute to the adaptation of an existing self-management program [30], combined with ehealth, to the experiences and needs of employees with CANS. Moreover, the results may also be useful for healthcare professionals and management aiming to support these employees. As part of the needs assessment (step one in the IM protocol) we also reviewed the Dutch multidisciplinary guideline for nonspecific CANS [21] and conducted focus groups with intervention and ehealth experts. We expect that focus groups with experts can have a surplus value. By comparing experiences of clients and interventionists we are able to analyze in which way the ehealth and self-management program needs to fit existing intervention strategies and which delivery strategies should be used. The results of these latter focus groups, and the results of the development of the intervention following the IM protocol, will be published in two separate forthcoming articles.

## Conclusions

In conclusion, employees suffering from CANS have to deal with their complaints in their daily life and at work. Several recurring problem areas have been identified and the results endorse the multifactorial origin of CANS. In general, participants experience problems similar to those of employees with other types of complaints or chronic diseases. These problems are related to their illness, insufficient awareness of possibilities to influence and manage their complaints themselves, inadequate communication with supervisors, and lacking adaptations at the workplace.

## Abbreviations

CANS: Complaints of the arm, neck or shoulder; IM: Intervention mapping; WMO: Dutch law on ‘Medical Research involving Human Subjects’.

## Competing interests

The authors do not have any competing interests.

## Authors’ contributions

YH, JE, JBS and RN developed the initial study proposal and acquired funding for the study. All authors developed the question guide. NH moderated the focus group sessions. All authors provided valuable input to this design paper, which was drafted by NH. All authors commented on the draft versions. All authors have read and approved the final manuscript.

## Pre-publication history

The pre-publication history for this paper can be accessed here:

http://www.biomedcentral.com/1471-2474/15/141/prepub

## Supplementary Material

Additional file 1 Question guide for the focus group sessions. Click here for file

## References

[B1] Huisstede BM Wijnhoven HA Bierma-Zeinstra SM Koes BW Verhaar JA Picavet S Prevalence and characteristics of complaints of the arm, neck, and/or shoulder (CANS) in the open population Clin J Pain 2008 24 253 259 10.1097/AJP.0b013e318160a8b4 18287832

[B2] Huisstede BM Bierma-Zeinstra SM Koes BW Verhaar JA Incidence and prevalence of upper-extremity musculoskeletal disorders. A systematic appraisal of the literature BMC Musculoskelet Disord 2006 7 7 10.1186/1471-2474-7-7 16448572 PMC1434740

[B3] van Tulder M Malmivaara A Koes B Repetitive strain injury Lancet 2007 369 1815 1822 10.1016/S0140-6736(07)60820-4 17531890

[B4] Karels CH Bierma-Zeinstra SM Verhagen AP Koes BW Burdorf A Sickness absence in patients with arm, neck and shoulder complaints presenting in physical therapy practice: 6 months follow-up Man Ther 2010 15 476 481 10.1016/j.math.2010.04.001 20570208

[B5] Van Eerd D Beaton D Cole D Lucas J Hogg-Johnson S Bombardier C Classification systems for upper-limb musculoskeletal disorders in workers: a review of the literature J Clin Epidemiol 2003 56 925 936 10.1016/S0895-4356(03)00122-7 14568622

[B6] Keijsers E Feleus A Miedema HS Koes BW Bierma-Zeinstra SM Psychosocial factors predicted nonrecovery in both specific and nonspecific diagnoses at arm, neck, and shoulder J Clin Epidemiol 2010 63 1370 1379 10.1016/j.jclinepi.2010.01.015 20430579

[B7] Martimo KP Shiri R Miranda H Ketola R Varonen H Viikari-Juntura E Self-reported productivity loss among workers with upper extremity disorders Scand J Work Environ Health 2009 35 301 308 10.5271/sjweh.1333 19471843

[B8] Harrington CB Siddiqui A Feuerstein M Workstyle as a predictor of pain and restricted work associated with upper extremity disorders: a prospective study J Hand Surg Am 2009 34 724 731 19345878 10.1016/j.jhsa.2008.12.012

[B9] Hooftman W Klein Hesselink J van Genabeek J Wiezer N Willems D Arbobalans 2010: Kwaliteit van de arbeid, effecten en maatregelen in Nederland [Quality of work, effects and measures in the Netherlands] (Dutch) 2011 Hoofddorp: Van der Ridder druk en print. the Netherlands

[B10] Blatter BHI Van den Bossche S Kraan K Van den Heuvel S Gezondheidsschade en kosten door RSI en psychosociale arbeidsbelasting 2006 Den Haag: Ministerie van Sociale Zaken en Werkgelegenheid

[B11] van Eijsden-Besseling MD Peeters FP Reijnen JA de Bie RA Perfectionism and coping strategies as risk factors for the development of non-specific work-related upper limb disorders (WRULD) Occup Med (Lond) 2004 54 122 127 10.1093/occmed/kqh003 15020731

[B12] Feleus A Bierma-Zeinstra SM Miedema HS Verhagen AP Nauta AP Burdorf A Verhaar JA Koes BW Prognostic indicators for non-recovery of non-traumatic complaints at arm, neck and shoulder in general practice–6 months follow-up Rheumatology (Oxford) 2007 46 169 176 10.1093/rheumatology/kel164 16799176

[B13] Bongers PM IJmker S van den Heuvel S Blatter BM Epidemiology of work related neck and upper limb problems: psychosocial and personal risk factors (part I) and effective interventions from a bio behavioural perspective (part II) J Occup Rehabil 2006 16 279 302 16850279 10.1007/s10926-006-9044-1

[B14] Staal JB de Bie RA Hendriks EJ Aetiology and management of work-related upper extremity disorders Best Pract Res Clin Rheumatol 2007 21 123 133 10.1016/j.berh.2006.09.001 17350548

[B15] Meijer EM Sluiter JK Frings-Dresen MH Is workstyle a mediating factor for pain in the upper extremity over time? J Occup Rehabil 2008 18 262 266 10.1007/s10926-008-9145-0 18688697 PMC2522379

[B16] Nicholas RA Feuerstein M Suchday S Workstyle and upper-extremity symptoms: a biobehavioral perspective J Occup Environ Med 2005 47 352 361 10.1097/01.jom.0000158705.50563.4c 15824626

[B17] Martimo KP Shiri R Miranda H Ketola R Varonen H Viikari-Juntura E Effectiveness of an ergonomic intervention on the productivity of workers with upper-extremity disorders–a randomized controlled trial Scand J Work Environ Health 2010 36 25 33 10.5271/sjweh.2880 19960145

[B18] Esmaeilzadeh S Ozcan E Capan N Effects of ergonomic intervention on work-related upper extremity musculoskeletal disorders among computer workers: a randomized controlled trial Int Arch Occup Environ Health 2014 2014 87 73 83 23263694 10.1007/s00420-012-0838-5

[B19] Verhagen AP Bierma-Zeinstra SM Feleus A Karels C Dahaghin S Burdorf L de Vet HC Koes BW Ergonomic and physiotherapeutic interventions for treating work-related complaints of the arm, neck or shoulder in adults. A Cochrane systematic review Eura Medicophys 2007 43 391 405 17921965

[B20] Hoe VC Urquhart DM Kelsall HL Sim MR Ergonomic design and training for preventing work-related musculoskeletal disorders of the upper limb and neck in adults Cochrane Database Syst Rev 2012 8 CD008570 22895977 10.1002/14651858.CD008570.pub2 PMC6486299

[B21] Beumer A de Bie RA ten Cate A Multidisciplinaire richtlijn aspecifieke Klachten Arm, Nek en/of Schouders [Guideline in non-specific complaints of the arm, neck and/or shoulder] (Dutch) 2012 Amersfoort: Koninklijk Nederlands Genootschap voor Fysiotherapie

[B22] Cole DC Van Eerd D Bigelow P Rivilis I Integrative interventions for MSDs: nature, evidence, challenges & directions J Occup Rehabil 2006 16 359 374 16933147 10.1007/s10926-006-9032-5

[B23] Bernaards CM Ariens GA Simons M Knol DL Hildebrandt VH Improving work style behavior in computer workers with neck and upper limb symptoms J Occup Rehabil 2008 18 87 101 10.1007/s10926-007-9117-9 18175072

[B24] Bernaards CM Ariens GA Knol DL Hildebrandt VH The effectiveness of a work style intervention and a lifestyle physical activity intervention on the recovery from neck and upper limb symptoms in computer workers Pain 2007 132 142 153 10.1016/j.pain.2007.06.007 17768009

[B25] Lorig KR Mazonson PD Holman HR Evidence suggesting that health education for self-management in patients with chronic arthritis has sustained health benefits while reducing health care costs Arthritis Rheum 1993 36 439 446 10.1002/art.1780360403 8457219

[B26] Lorig KR Holman H Self-management education: history, definition, outcomes, and mechanisms Ann Behav Med 2003 26 1 7 12867348 10.1207/S15324796ABM2601_01

[B27] Lebensohn P Dodds S Benn R Brooks AJ Birch M Cook P Schneider C Sroka S Waxman D Maizes V Resident wellness behaviors: relationship to stress, depression, and burnout Fam Med 2013 45 541 549 24129866

[B28] Barlow J Wright C Sheasby J Turner A Hainsworth J Self-management approaches for people with chronic conditions: a review Patient Educ Couns 2002 48 177 187 10.1016/S0738-3991(02)00032-0 12401421

[B29] Newman S Steed L Mulligan K Self-management interventions for chronic illness Lancet 2004 364 1523 1537 10.1016/S0140-6736(04)17277-2 15500899

[B30] Detaille SI Building a self-management program for workers with a chronic somatic disease 2012 PhD Dissertation. Nijmegen: Ipskamp Drukkers B.V.

[B31] Detaille SI Heerkens YF Engels JA van der Gulden JW van Dijk FJ Effect evaluation of a self-management program for dutch workers with a chronic somatic disease: a randomized controlled trial J Occup Rehabil 2013 23 189 199 10.1007/s10926-013-9450-0 23690087

[B32] Bartholomew LK Parcel GS Kok G Intervention mapping: a process for developing theory- and evidence-based health education programs Health Educ Behav 1998 25 545 563 10.1177/109019819802500502 9768376

[B33] Gosling CM Forbes AB Gabbe BJ Health professionals’ perceptions of musculoskeletal injury and injury risk factors in Australian triathletes: a factor analysis Phys Ther Sport 2013 14 207 212 10.1016/j.ptsp.2012.09.004 23177357

[B34] Krueger RA Casey MA Focus Groups. A Practical Guide for Applied Research 2009 Thousand Oaks, California: Sage Publications, Inc

[B35] Hsieh HF Shannon SE Three approaches to qualitative content analysis Qual Health Res 2005 15 1277 1288 10.1177/1049732305276687 16204405

[B36] Elo S Kyngas H The qualitative content analysis process J Adv Nurs 2008 62 107 115 10.1111/j.1365-2648.2007.04569.x 18352969

[B37] Downe-Wamboldt B Content analysis: method, applications, and issues Health Care Women Int 1992 13 313 321 10.1080/07399339209516006 1399871

[B38] Karlqvist L Wigaeus TE Hagberg M Hagman M Toomingas A Self-reported working conditions of VDU operators and associations with musculoskeletal symptoms: a cross-sectional study focussing on gender differences Int J Ind Ergon 2003 30 277 294

[B39] Brandt LP Andersen JH Lassen CF Kryger A Overgaard E Vilstrup I Mikkelsen S Neck and shoulder symptoms and disorders among Danish computer workers Scand J Work Environ Health 2004 30 399 409 10.5271/sjweh.828 15529803

[B40] Barbour RS Checklists for improving rigour in qualitative research: a case of the tail wagging the dog? BMJ 2001 322 1115 1117 10.1136/bmj.322.7294.1115 11337448 PMC1120242

[B41] Detaille SI Haafkens JA Hoekstra JB van Dijk FJ What employees with diabetes mellitus need to cope at work: views of employees and health professionals Patient Educ Couns 2006 64 183 190 10.1016/j.pec.2005.12.015 16469470

[B42] Detaille SI Haafkens JA van Dijk FJ What employees with rheumatoid arthritis, diabetes mellitus and hearing loss need to cope at work Scand J Work Environ Health 2003 29 134 142 10.5271/sjweh.715 12718499

[B43] Carnes D Homer KE Miles CL Pincus T Underwood M Rahman A Taylor SJ Effective delivery styles and content for self-management interventions for chronic musculoskeletal pain: a systematic literature review Clin J Pain 2012 28 344 354 10.1097/AJP.0b013e31822ed2f3 22001667

[B44] Nolte S Osborne RH A systematic review of outcomes of chronic disease self-management interventions Qual Life Res 2013 22 1805 1816 10.1007/s11136-012-0302-8 23111571

[B45] Du S Yuan C Xiao X Chu J Qiu Y Qian H Self-management programs for chronic musculoskeletal pain conditions: a systematic review and meta-analysis Patient Educ Couns 2011 85 e299 e310 10.1016/j.pec.2011.02.021 21458196

[B46] Gustavsson C Denison E von Koch L Self-management of persistent neck pain: a randomized controlled trial of a multi-component group intervention in primary health care Eur J Pain 2010 14 630 e631–630 e611 19939717 10.1016/j.ejpain.2009.10.004

