# Supplementary material for: Experiences of employees with arm, neck or shoulder complaints: a focus group study
Source: BMC Musculoskelet Disord. 2014 Apr 29;15:141. doi: 10.1186/1471-2474-15-141 (PMC4021174; doi:10.1186/1471-2474-15-141)
Supplement: Additional file 1 — Question guide for the focus group sessions. [file 1471-2474-15-141-S1.docx]

**Appendix: Question guide for the focus group sessions**

**Introduction (5 minutes)**

**Introduction of participants (10 minutes)**

- Name, work, what do you like about your work?
- What complaints do you have and how long have you had them?

**Complaints (20 minutes)**

- What have you done to decrease your complaints?
- Have you searched for information about your complaints?
- Do you still have a need for more information?
- What do you think are the causes of your complaints?

**Work (25 minutes)**

- Which problems do you experience at work due to your complaints?
- How do you deal with these problems?
- Are there any problems about which you would like to have more information or training?

***Pause (10 minutes)***

**Facilities (10 minutes)**

- There are various facilities within the institution for CANS. Which facilities do you use and which not? Why (not)?
- Which facilities do you need in order to continue your work, despite your complaints?

**Social support (5 minutes)**

- Do you receive enough support from your colleagues?
- Do you receive enough support from your family?
- Do you receive enough support from your superiors?

**Asking for help (5 minutes)**

- If needed, do you ask for help? With whom? Why not?
- Which healthcare professionals did you have contact with for your complaints?

**Communication (5 minutes)**

- With whom do you communicate about your complaints
- What problems do you experience in the communication about your complaints with colleagues, your manager(s) and healthcare professionals?

**Lifestyle (10 minutes)**

- Do you participate in sport activities? If yes, what kind of activities?
- Do you perform exercises for your complaints?
- Do you need more information about sport activities and/or exercises?

**Closure (10 minutes)**

- What would you like to learn in a self-management program for employees suffering from CANS?
- What kind of information would you like to obtain?
- What do you consider as most important when designing a self-management program for employees suffering from CANS?
- Is there anything else you would like to say?
